# Effects of Dietary *Eucommia ulmoides* Leaf Extract Supplementation on Growth Performance, Meat Quality, Antioxidant Capacity, and Lipid Metabolism of Finishing Pigs

**DOI:** 10.3390/antiox13030320

**Published:** 2024-03-06

**Authors:** Mengmeng Han, Yunju Yin, Saiming Gong, Hanjing Shi, Qilong Li, Xiao Lian, Yehui Duan, Fengna Li, Qiuping Guo

**Affiliations:** 1 Hunan Provincial Key Laboratory of Animal Nutritional Physiology and Metabolic Process, Key Laboratory of Agro-Ecological Processes in Subtropical Region, Institute of Subtropical Agriculture, Chinese Academy of Sciences, Hunan Provincial Engineering Research Center for Healthy Livestock and Poultry Production, Changsha 410125, China; hanmengmeng21@mails.ucas.ac.cn (M.H.); yinyinyj1124@163.com (Y.Y.); gongsaiming@foxmail.com (S.G.); shihj1015@163.com (H.S.); liqilong23@mails.ucas.ac.cn (Q.L.); 20211200637@csuft.edu.cn (X.L.); duanyehui@isa.ac.cn (Y.D.); lifengna@isa.ac.cn (F.L.); 2 College of Advanced Agricultural Sciences, University of Chinese Academy of Sciences, Beijing 100049, China; 3 College of Life Sciences, Hunan Normal University, Changsha 410128, China; 4 National Engineering Laboratory for Rice and By-Product Deep Processing, Central South University of Forestry and Technology, Changsha 410004, China

**Keywords:** *Eucommia ulmoides*, pork quality, amino acids, antioxidant status, lipid metabolism

## Abstract

This study aimed to investigate the effects of dietary *Eucommia ulmoides* leaf extract (ELE) on meat quality, antioxidant capacity, and lipid metabolism in finishing pigs. A total of 240 “Duroc × Landrace × Yorkshire” crossbred pigs with an initial weight of 74.70 ± 0.77 kg were randomly assigned to two groups: control group and 0.2% ELE group, with each group containing 10 replicates of 12 pigs per pen (half barrows and half gilts). The data showed dietary 0.2% ELE supplementation did not affect growth performance but tended to reduce the backfat thickness of the finishing pigs (*p* = 0.07). ELE diets increased pH value (*p* < 0.05) and meat color score (*p* = 0.01) and decreased 45 min L* value (*p* < 0.05), 24 h L* value (*p* = 0.01), pressurization loss (*p* = 0.01), and 24 h drip loss (*p* < 0.05) in *longissimus dorsi* (LD) muscle, accompanied by an increased (*p* < 0.05) proportion of monounsaturated fatty acids (MUFA) and decreased polyunsaturated fatty acids (PUFA) (*p* = 0.06) and n-6/n-3 PUFA ratio (*p* = 0.05) compared to controls. In addition, ELE supplementation increased inosine monophosphate (IMP) (*p* = 0.01), sweet amino acids (AAs) (*p* < 0.05), and total free AA content (*p* = 0.05) in LD. Meanwhile, increased activity of glutathione peroxidase (*p* < 0.05) and superoxide dismutase (*p* < 0.01) in both serum and LD muscle and decreased malondialdehyde content (*p* < 0.01) in LD muscle were detected with ELE treatment. Moreover, pigs fed ELE had a higher total protein (*p* < 0.01), albumin (*p* < 0.05), and high-density lipoprotein cholesterol (*p* < 0.05) and a lower total cholesterol (*p* < 0.01) and triacylglycerols (*p* = 0.06) in serum. Consistently, significant effects of dietary ELE were observed on the relative mRNA expression of lipid metabolism in the backfat and the LD muscle, respectively. ELE attenuated lipogenic processes in backfat, decreasing the relative expression of acetyl-CoA carboxylase and upregulating the relative expression of adipose triacyl glyceride lipase, carnitine palmitoyl transferase 1B, and fatty acid-binding protein 4 (*p* < 0.05). ELE also decreased the relative expression of CCAAT/enhancer-binding protein α (*p* < 0.05), fatty acid translocase (*p* < 0.05), carnitine palmitoyl transferase 1B (*p* < 0.01), and adipose triacyl glyceride lipase (*p* < 0.05) in LD muscle (*p* < 0.05). More specifically, lipogenesis appeared to be inhibited in both LD muscle and backfat, with the difference being that lipolysis was enhanced in backfat and inhibited in LD muscle. In conclusion, dietary ELE supplementation can potentially enhance carcass traits, sensory quality, and nutritional value of pork without negatively affecting intramuscular fat content. The underlying mechanism for these positive effects may be linked to the alterations in lipid metabolism and increased antioxidant capacity induced by ELE.

## 1. Introduction

Pork is one of the most consumed meats worldwide [1]. However, the excessive pursuit of growth performance and lean meat percentage in pigs has led to a deterioration of pork quality [2], becoming a shared concern among consumers and the pig industry worldwide [3]. Consequently, increasing attention has been paid to pork’s safety, sensory quality, and nutritional value. Tenderness, intramuscular fat (IMF), and various flavor compounds are decisive in determining its sensory quality [4,5]. The nutritional value of pork is reflected in its ability to provide humans with rich nutrients such as protein, fatty acids, minerals, and vitamins [6]. It has been demonstrated in numerous studies that dietary supplements such as plant extracts [7,8], fatty acids [9], amino acids [10], and minerals [11] can enhance the quality of pork. The antioxidant capacity of pig body tissues is positively correlated with their own health and meat quality, influencing sensory qualities such as lipid and protein oxidation levels and meat color and juiciness in pork [8,12]. Lipid peroxidation, caused by the overproduction of reactive oxygen species (ROS) (such as O_2_^•−^, H_2_O_2_ and ^•^OH) [13], damages the integrity of muscle cell membranes and ultimately leads to reduced water-binding capacity and deteriorated meat color [2]. In addition, the composition of amino acids and fatty acids significantly influences meat flavor development [14]. IMF deposition is the result of increased fat synthesis and degradation [14,15].

Recently, more and more reports have mentioned that some natural or synthetic nutrients can improve the meat quality and antioxidant capacity of livestock and poultry [6,10,12,13]. *Eucommia ulmoides* Oliver, a unique species of the *Eucommia* family in China, belongs to the *Eucommia* genus and has been used in the field of traditional Chinese medicine for nearly two thousand years [16]. *Eucommiae folium* is rich in lignans, iridoids, flavonoids, polysaccharides, and other active ingredients [17], exhibiting pharmacological effects such as antihypertensive, blood lipid regulation, hypoglycemic, anti-inflammatory, antioxidant, anti-tumor, and liver protection [18,19]. Recently, reports have highlighted the ability of *Eucommia* extract to improve meat quality in chicken [20], lamb [21], and fish [22]. Our previous studies reported that dietary supplementation with 0.1–0.2% ELE is the optimal range to reduce fat deposition in pig backfat tissue [23], and we also reported that 0.04% chlorogenic acid (CGA) improved muscle amino acid composition in finishing pigs [24]. Given the available studies and our accumulation of previous work, we hypothesize that dietary 0.2% ELE supplementation may change the meat quality traits of finishing pigs.

Therefore, this study aimed to evaluate the effects of dietary 0.2% ELE supplementation on growth performance, meat quality, muscle fatty acid, amino acid composition, antioxidant capacity, and lipid metabolism in finishing pigs. This study will provide experimental evidence for the development and utilization of ELE as a natural feed additive to improve the meat quality of finishing pigs.

## 2. Materials and Methods

### 2.1. Animals and Diets

A total of 240 healthy “Duroc × Landrace × Yorkshire” crossbred finishing pigs with an average body weight of 74.70 ± 0.77 kg and 133 days old were chosen for the study. According to the recommendation of the National Research Council [25], these pigs were fed a diet based on corn and soybean meal to meet the nutritional requirements of pigs weighing 61–90 and 91–120 kg. The pigs were randomly assigned to two groups with 10 replicates (pens) per group and 12 pigs (half barrows and half gilts) per pen. Pigs in the control group were fed a basal diet, while the experimental group was fed diets supplemented with 0.2% ELE at the expense of wheat bran. All pigs were provided with ad libitum access to feed and water throughout the entire 60-day experimental period. The composition and nutrient levels of the basal diet are presented in Table 1. ELE was supplied by Zhangjiajie Hengxing Biotechnology Co., Ltd. (Zhangjiajie, China) and contained active ingredients consisting of 2.8% Moisture, 3.89% Ash, 5% CGA, 8% *Eucommia* flavonoids, and 20% *Eucommia* polysaccharide. ELE was mainly prepared through the following steps: First, the fresh leaves were dried to control the water content < 13% and then crushed. The crushed leaves were added to the multifunctional dynamic extraction tank, the ratio of water to leaves was controlled at 1:6–8, the pH value was adjusted to 5–6, the temperature was heated to 65–80 °C, and the leaves were extracted for 1 h in the forced reflux cycle, with a total of 2–3 extraction cycles. After the extraction, the extraction liquid was quickly cooled to 20~25 °C and placed in a sedimentation tank for 3~6 h, then centrifuged to remove the sedimentation. The centrifuged material solution was concentrated at 60 °C in a vacuum, and the concentrated *Eucommia* extract leaf was dried to obtain the powdered finished product.

### 2.2. Sample Collection

At the end of the feeding trial and a fasting period of 12 h, the individual weight was measured. Then, one pig with average body weight was selected for slaughter per replicate (half barrows and gilts). Blood samples were collected by inferior vena cava puncture into 10 mL tubes and coagulated at room temperature. The serum was immediately separated by centrifugation (3000× *g*, 15 min, 4 °C) and stored at −80 °C until use. The pigs were stunned electrically (250 V, 0.5 A, 5–6 s), which was followed by exsanguination and evisceration. After being scalded (60–65 °C for 45–60 s), dehaired, and eviscerated, the hot carcass weight was recorded for the calculation of dressing percentage. The *longissimus dorsi* (LD) muscle samples from the right side of the carcass were rapidly excised to measure 45 min and 24 h sensory indexes of meat quality. About 200 g of LD muscle sample from the left side of the carcass at the 10th rib was frozen at −20 °C for measurements of muscle chemical, amino acid, and fatty acid composition. In addition, approximately 1 cm^3^ of LD muscle sample from the left side of the carcass at the 10th rib was frozen in liquid nitrogen and used for RNA extraction and enzyme activity assessment, as well as malondialdehyde (MDA) content measurement.

### 2.3. Growth Performance, Carcass Traits, and Meat Quality

The weights of the pigs in each pen were recorded at the experiment’s start and end to determine the average daily weight gain (ADG) using their initial and final weights. Moreover, the total feed intake for each pen during the entire phase was recorded to estimate the average daily feed intake (ADFI), from which the feed-to-gain ratio (F/G) was calculated.

Hot carcass weight was immediately recorded after slaughter and subsequently used to calculate carcass yield. Backfat depth was measured at the first rib, the 6th to 7th rib, and the last lumbar vertebra, and loin eye height and width (loin eye area (cm^2^) = loin eye height (cm) × width (cm) × 0.7) were also recorded. A portable pH meter (Matthau’s pH Star, Germany) was used to determine pH values on the LD muscle at 45 min and 24 h postmortem, calibrated with pH 4.6 and 7.0 buffers at the beginning of each measurement day according to the method described by Yin et al. [10]. Meat color, including lightness (L*), redness (a*), and yellowness (b*) values, was measured at 45 min and 24 h postmortem using a handheld colorimeter (CR-410, Kinica Minolta Sensing Inc., Osaka, Japan). Before each measurement, the instrument was calibrated with a standard white plate. Drip loss was calculated according to the following formula: drip loss (%) = [(initial weight − final weight)/initial weight] × 100. The cooking loss was calculated using the difference between the initial and final cooking weights. Specifically, the method involved cooking the meat in a water bath at 75 °C until the internal temperature reached 70 °C. After cooling for 30 min, the cooked samples were blotted dry and weighed. Pressurization loss was measured by pressure instrument platform (350 N 5 min) as recently described by Zheng et al. in detail [26]. The meat color score and marbling score using the National Pork Producers Council (NPPC) scale of the LD were evaluated on the cut surface at the intercostal space between the 10th and 11th ribs [27].

### 2.4. Muscle Chemical Composition

The IMF, dry matter (DM), and crude protein (CP) contents were determined using the methods of the Association of Analytical Chemists [28]. Inosine monophosphate (IMP) was measured according to the methods we described previously [10].

### 2.5. Serum Biochemical Indexes

Serum biochemical indexes, including total protein (TP), albumin (ALB), urea nitrogen (BUN), blood glucose (GLU), triacylglycerols (TAG), total cholesterol (TC), low-density lipoprotein cholesterol (LDL-C), and high-density lipoprotein cholesterol (HDL-C) were determined using a Roche automatic biochemical analyzer (Cobas c311; F. Hoffmann-La Roche Ltd., Basel, Switzerland) and commercial kits (F. Hoffmann-La Roche Ltd., Basel, Switzerland).

### 2.6. Serum and Longissimus Dorsi Muscle Antioxidant Indexes

Fresh LD muscle samples (stored at −80 °C) were put into Eppendorf tubes and homogenized with grinding beads, diethylpyrocarbonate-treated water, and protease inhibitors. The activities of total superoxide dismutase (SOD), glutathione peroxidase (GSH-Px), and catalase (CAT) and the content of malondialdehyde (MDA) were measured with commercial kits (Changsha Aoji Biotechnology Co., Ltd., Changsha, China).

### 2.7. Fatty Acid Composition

Gas chromatography was used to determine the fatty acid composition of LD muscle. A total of approximately 0.5 g of LD muscle samples after freeze-drying was weighed, placed in a 50 mL centrifuge tube, and sealed with 5 mL of benzene-petroleum ether (*v*:*v* = 1:1 mix) solvent for 24 h. After the extraction, a 5 mL potassium hydroxide–methanol solution (0.4 mol/L) was added to the sample, and then the sample was shaken on the vortex mixer for 3 min. After 30 min, ultrapure water was added in separate layers to the test tube. After centrifugation, the upper-layer solution was removed, and a certain amount of anhydrous sodium sulfate was added to absorb water. Finally, the sample was filtered by a 0.22-micron filter membrane (NYL), and the obtained fatty acid methyl esters were analyzed using an Agilent 6890 gas chromatograph with a flame ionization detector (Agilent Technologies, Santa Clara, CA, USA). A CP-Sil 88 fused silica open tubular capillary column (100 m, 0.25 nm; Chrompack, Boeckten, Switzerland) was used. The fatty acid composition is presented as a percentage of total fatty acids. The following formula was used to calculate the lipid quality indices [29]: Atherosclerosis index (AI) = (C12:0 + (4 C14:0) + C16:0)/(n-3 PUFA + n-6 PUFA + MUFA). Index of thrombogenicity (TI): TI = (C14:0 + C16:0 + C18:0)/((0.5 × C18:1) + (0.5 × other MUFA) + (0.5 × Σn-6 PUFA) + (3 × Σn-3 PUFA) + Σn-3 PUFA/Σn-6PUFA). Ratio of hypocholesterolemic and hypercholesterolemic fatty acids (h/H): h/H = (C18:1n-9 + C18:2n-6 + C18:3n-3)/(C12:0 + C14:0 + C16:0).

### 2.8. Amino Acid Composition and Taste Activity Value (TAV)

Based on previous research [24], the free amino acid profile in the LD muscles was determined using a modified high-performance liquid chromatography (HLPC) instrument. Approximately 2.5 g of fresh meat samples were weighed sequentially and homogenized completely with 10 mL of 0.01 M HCl. The mixture was stirred in an ultrasonic bath for 30 min, and then the supernatant was transferred to a 25 mL volumetric flask and filtered by dilution with distilled water. The filtrate (2 mL) was homogenized with an equal volume of hexane solution, and the subnatant (1 mL) was separated, followed by homogenization of the subnatant with an equal volume of 0.8% sulfosalicylic acid solution and incubated overnight at 4 °C. The mixture was centrifuged at 10,000× *g* for 10 min. Subsequently, the supernatant was filtered through a 0.22 mm microfilter. Finally, the filtrate was labeled with iTRAQ reagent (AA 45/32 kit; Applied Biosystems, Foster City, CA, USA) according to the manufacturer’s recommendations and analyzed using the same high-performance liquid chromatography method described above. The TAV was calculated according to the following formula by Liu et al. [30]: TAV = concentration of the component in the sample (mg/100 g)/threshold value (mg/100 g).

### 2.9. Relative mRNA Expression

Total RNA was extracted from *longissimus dorsi* and backfat using Trizol reagent (15596-026, Invitrogen, MA, USA). Then, Nano Drop ND-1000 (Thermo Scientific, Waltham, MA, USA) was used to determine the concentration and quality of total RNA, and the RNA reverse transcription kit (Takara Biotechnology Co., Ltd., Dalian, China) was used to synthesize cDNA according to the instructions. The expression levels of lipid metabolism-related genes were determined by real-time PCR using the synthesized cDNA as a template. The primers used for RT-qPCR (Table 2) were designed using Primer premier 5.0 software; *GAPDH* was used as an internal control, and the relative expression of the target gene was calculated by 2^−(ΔΔCt)^ methods [31].

### 2.10. Statistical Analysis

The original data were processed by Excel 2018 (Microsoft Corporation, Redmond, WA, USA). Growth performance data were analyzed by the Mixed model, with diet as the fixed and pen as the random effect. The other data were tested by Student’s *t* test by Statistical Package for the Social Sciences (SPSS) version 26.0 software. The pen of pigs served as the experimental unit. All data are presented as mean ± SEM. Differences were considered statistically significant at *p* ≤ 0.05 and a trend at 0.05 < *p* < 0.10.

## 3. Results

### 3.1. Growth Performance and Carcass Characteristics

As shown in Table 3, no significant difference was observed in the final body weight, ADFI, ADG, F/G, carcass yield, carcass length, loin-eye area, liver, and perirenal fat weight (*p* > 0.05). Compared to the control group, dietary 0.02% ELE supplementation tended to decrease the backfat thickness (*p* = 0.07) of the finishing pigs.

### 3.2. Meat Quality

The meat quality of the pig is shown in Table 4. No effect (*p* > 0.05) of dietary 0.2% ELE was detected on cooking loss, marbling score, a* value, 24 h b* value, and shear force. However, compared to the control group, dietary 0.2% ELE supplementation resulted in an increased pH value (*p* < 0.05) and decreased 45 min L* value (*p* < 0.05) and 24 h L* value (*p* = 0.01). In addition, pressurization loss (*p* = 0.01) and 24 h drip loss (*p* < 0.05) in 0.2% ELE were lower than those in the control group. Moreover, dietary 0.2% ELE supplementation significantly increased meat color score (*p* = 0.01).

### 3.3. Muscle Chemical Composition

There were no differences in DM, IMF, and CP (*p* > 0.05) between different groups of finishing pigs (Table 5), but increased IMP levels in the ELE group were observed (*p* = 0.01).

### 3.4. Serum Biochemical Indexes

Serum metabolite concentrations in the two dietary groups are shown in Table 6. Dietary 0.2% ELE supplementation did not affect the contents of BUN, GLU, and LDL (*p* > 0.05) while increasing the content of TP (*p* < 0.01), ALB, and HDL (*p* < 0.05), and it decreased TC (*p* < 0.01) and tended to decrease TAG (*p* = 0.06) levels.

### 3.5. Antioxidant Enzyme Activities and MDA Content

As indicated in Table 7, ELE dietary supplementation at 0.2% significantly increased serum GSH-PX and SOD activity (*p* < 0.01). Similarly, dietary 0.2% ELE supplementation did not affect (*p* > 0.05) the LD muscle CAT activity but significantly increased the LD muscle GSH-PX (*p* < 0.05) and SOD (*p* < 0.01) activity. LD muscle MDA content was lower in the ELE group than in the control group (*p* < 0.01).

### 3.6. Fatty Acid Composition

Table 8 shows the fatty acid composition of the LD of pigs. Compared with the control treatment, 0.2% ELE supplementation increased the concentrations of C14:0 (*p* < 0.05), C18:1 cis-9 (*p* < 0.05), and C20:1 (*p* = 0.05) and decreased those of C18:0 (*p* = 0.05), C18:2n-6 (*p* = 0.09), C18:3n-6 (*p* < 0.05), C20:3n-6 (*p* < 0.05), C20:4n-6 (*p* = 0.05), and C24:0 (*p* < 0.05) but had no significant effects on total unsaturated fatty acids (UFA) or total saturated fatty acids (SFA) in LD muscle (*p* > 0.05). In addition, 0.2% ELE supplementation significantly increased (*p* < 0.05) the content of MUFA compared to the controls and tended to decrease (*p* = 0.06) the content of PUFA. Notably, a lower n-6/n-3 PUFA ratio was observed in the 0.2% ELE group (*p* = 0.05), but no significant difference was observed for n-3 PUFA, SFA/PUFA, AI, TI, or h/H between the two groups (*p* > 0.05).

### 3.7. Free Amino Acid Composition of Muscle and Taste Activity Value

The composition of free amino acids in LD muscle is shown in Table 9, and a total of 18 amino acids were analyzed. In detail, the taste activity values (TAVs) of aspartic (*p* < 0.05), alanine (*p* = 0.05), and serine (*p* = 0.05) were significantly increased with ELE supplementation. In addition, 0.2% ELE supplementation improved the TAVs of isoleucine (*p* < 0.01) and histidine (*p* = 0.01) and reduced the TAV of arginine (*p* = 0.01) compared with CON. Furthermore, dietary 0.2% ELE significantly increased tyrosine (*p* = 0.01) content, while it tended to decrease cysteine (*p* = 0.08) concentration. In general, dietary ELE supplementation increased total AA (*p* = 0.05) and sweet AA (*p* < 0.05) content and tended to increase (*p* = 0.07) EAA and NEAA levels.

### 3.8. Lipid Metabolism-Related Gene mRNA Levels

**Figure 1:**
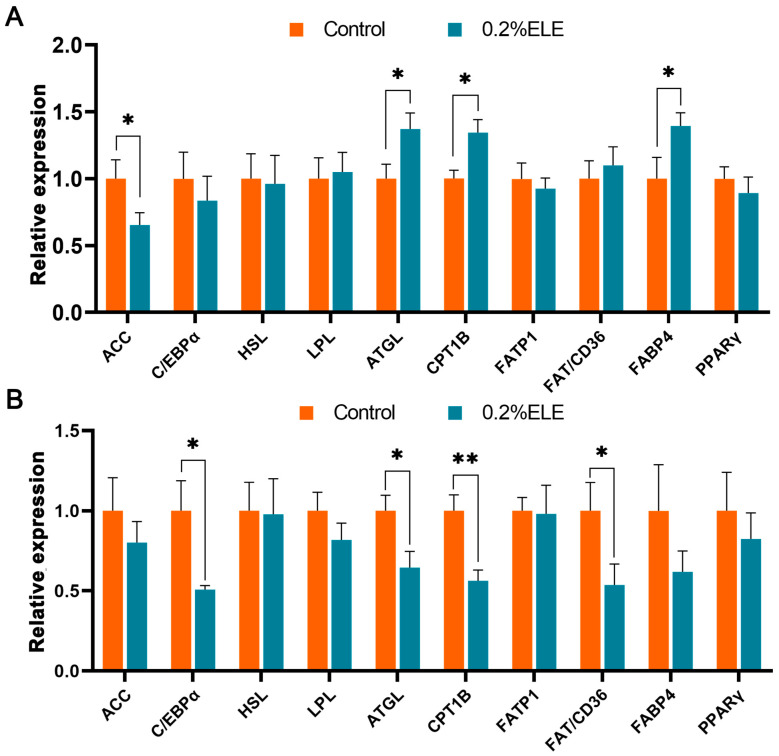
Effects of dietary ELE supplementation on mRNA levels of lipid metabolism-related genes in backfat (**A**) and *longissimus dorsi* muscle of finishing pigs (**B**). These genes include C/EBPα and PPARγ, which are key genes regulating lipid metabolism; ACC, which is related to lipid synthesis; HSL, LPL, ATGL, which are related to lipid degradation; CPT1B, which is related to fatty acid oxidation; FATP1, FAT/CD36, FABP4, which is related to fatty acid transport (*n* = 10). Data are presented as mean ± SEM, and significant differences are indicated by asterisks (* as *p* ≤ 0.05, ** as *p* < 0.01).

Figure 1A shows the expression of genes related to lipid metabolism in backfat. Dietary ELE supplementation significantly increased the relative mRNA levels of ATGL, CPT1B, and FABP4 and reduced the expression level of ACC (*p* < 0.05). No differences were observed in other genes of backfat between the two groups. For LD muscle, Figure 1B shows that ELE treatment significantly reduced the mRNA expression levels of C/EBPα (*p* < 0.05), FAT/CD36 (*p* < 0.05), CPT1B (*p* < 0.01), and ATGL (*p* < 0.05) genes.

## 4. Discussion

*Eucommia ulmoides* and its by-products are rich in phenols, ketones, polysaccharides, and amino acids [17]. Previous research has confirmed that ELE can improve the antioxidant capacity of animals, regulate lipid metabolism, and reduce inflammation [32,33,34]. These benefits are associated with improved health and enhanced meat quality of animals in livestock production [20,21,35]. Our study specifically investigated the impact of 0.2% ELE in the diet on finishing pigs. Surprisingly, we observed that there was no significant difference in final body weight, ADFI, ADG, and F/G of finishing pigs as an effect of dietary ELE supplementation. This finding suggests that ELE did not affect feed palatability. Interestingly, previous studies have shown that feeding piglets with an initial body weight of about 10 kg until the end of 115 kg with the same dose of ELE can increase the ADFI of growing pigs without significant effects on growth and feed intake [23]. However, it has been reported that diets supplemented with 3% or 5% *Eucommia ulmoides* leaf (EL) [36] or 0.08% polyphenolic extract (derived from EL) [37] have a significant effect on growth performance in finishing pigs. The reason for this inconsistency could possibly be attributed to differences in the growth phases of the experimental pigs, variations in feeding cycles, and the diverse modes of ELE supplementation. As we all know, lean meat percentage is an effective indicator of carcass quality, often estimated by carcass weight, 10th rib backfat thickness, and loin-eye area [38]. In this study, ELE supplementation showed a significant trend of decreasing backfat thickness of finishing pigs compared to the control group. This finding suggests that the bioactive substances in ELE may regulate lipid metabolism, as evidenced by the decrease in serum CHOL and TAG content in the ELE group. Moreover, ELE can prevent fat from accumulating by reducing the volume growth of adipocytes [23]. Thus, supplementation of feed with ELE can be considered to prevent excessive fat deposition during the rapid finishing period of pigs.

Meat color provides consumers with an intuitionistic basis for evaluating the quality of meat products. The depth of meat color is primarily caused by myoglobin (Mb) [39]. After slaughter, Mb physically combines with O_2_ in the air to form bright red oxymyoglobin (MbO_2_), which eventually oxidizes to metmyoglobin, resulting in a brown color [39,40]. In this study, some indicators of meat color, such as 45 min L* value, b* value, and 24 h L* value, were significantly lower than those of the control group, and the meat color score was significantly improved. Water-holding capacity is one of the most significant economic indicators of commercial meat, which is predominantly reflected by drip loss, pressurization loss, and cooking loss. Lipid peroxidation and rapid changes in pH value can destroy the integrity of the cell membrane, resulting in the decreased water-holding capacity of pork [2,41]. Excitingly, we found that the ELE supplementation diet increased pH_45min_ and pH_24h_ values and reduced drip loss and pressurization loss. When there is an imbalance between the antioxidant defenses and ROS, biological molecules such as lipids and proteins are damaged, resulting in decreased water-holding capacity, tenderness, and color of pork and the formation of MDA, aldehydes, ketones, and phenolic compounds, which significantly affect the safety and nutritional value of meat [2,42,43]. We know that the antioxidant enzyme system, including SOD, GSH-PX, and CAT, constitutes a critical component for maintaining cellular redox homeostasis, serving as the first line of antioxidant defenses [44]. SOD scavenges harmful superoxide free radicals (O_2_·^−^) in cells [45]. GSH-PX and CAT cooperate with SOD to reduce toxic peroxides into non-toxic hydroxyl compounds and promote the decomposition of H_2_O_2_, thus protecting the structure and function of cell membranes from the damage of peroxides [46]. MDA, a byproduct of lipid peroxidation, serves as an indicator of oxidative damage levels [47]. In this study, a significant increase in the activities of SOD and GSH-PX was observed in pig serum and muscle tissues treated with ELE, accompanied by a notable decrease in MDA content. Notably, there was an increasing trend in CAT activity in the serum. The reasonable explanation is that the digested and absorbed ELE can act as an exogenous antioxidant, increase the activity of SOD and GSH-PX, effectively reduce the level of lipid peroxidation caused by reactive ROS, reduce the concentration of MDA, better maintain the integrity of the cell membrane, and then improve the color and water-holding capacity of pork. In addition, Li et al. [48] found that feeding finishing pigs with CGA extracted from ELE in their diet can increase the activities of SOD and GSH-PX in muscle, decrease the MDA content, and effectively mitigate oxidative damage caused by oxidized corn oil. Similarly, Ding [49] reported that 0.3 g/kg of ELE can improve the antioxidant capacity of serum and liver in piglets. Our study demonstrates that improving muscle antioxidant properties and pH value may be the main reasons for higher water-holding capacity and better meat color in the ELE group.

The level of free amino acids in pork plays an important role in determining the nutritional value of meat, and the threshold and content of flavor amino acids will affect its flavor characteristics [9]. It has been shown that IMP and umami taste ratings are positively correlated [50]. This study showed that the ELE group had higher IMP levels, indicating an improvement in muscle flavor. Observations indicate that dietary supplementation with apple polyphenols can enhance the content of EAAs, NEAAs, and total AAs by boosting certain amino acid transporters in the LD muscle of finishing pigs [51]. Long et al. [51] discovered that honeysuckle leaf extract can improve the content of EAAs and total AAs in finishing pig LD muscle. We found that dietary ELE raised the concentrations of free EAAs, NEAAs, and total AAs in LD muscle and improved the nutritional value of pork, which was consistent with their findings. We speculated that these plant extracts increase the expression of amino acid transporters in muscle, thereby improving amino acid metabolism, with CGA playing a key role [24]. Amino acids are classified based on their flavor characteristics into umami, sweet, and bitter amino acids [52]. TAV, defined as the ratio of the concentration of flavor amino acids to their threshold value, can be used to determine the contribution of flavor compounds to the overall flavor of the sample. Notably, a higher TAV value correlates with a greater contribution to the sample’s overall flavor profile [30,53,54]. In our study, dietary ELE increased the content of sweet AAs and the TAV values of Ala, Ser, and Asp and decreased the TAV values of Arg and Met, indicating an improvement in muscle flavor. Additionally, we observed a significant increase in levels and TAV values for His and Ile and a significant decrease for Arg and Met upon dietary ELE supplementation, with no discernible change in the content of bitter AAs, indicating the absence of any deleterious effects on meat flavor. Some studies have introduced the concept of functional amino acids, which are not all essential amino acids but are usually involved in important metabolic pathways to promote animal growth, development and reproduction [55]. In addition to its role in protein metabolism, His is also an important prerequisite for the synthesis of carnosine and histamine [56]. Ile is a branched-chain amino acid that has a certain regulatory effect on the signal pathway of protein synthesis [57]. Previous reports suggest that Tyr regulates the dietary rhythms of rats and human appetite and may ameliorate obesity caused by poor dietary patterns [58]. Furthermore, our study revealed a significant upregulation of Tyr content upon ELE supplementation. In this study, when finishing pigs were fed 0.2% ELE, muscle contents of free essential amino acids, functional amino acids, sweet amino acids, and total amino acids increased, so we speculate that 0.2% ELE improves the amino acid nutritional structure and quality of pork.

Blood biochemical indexes can reflect the functional status, nutritional metabolism level, and growth performance of pig tissues and organs. The serum TP is an important marker for evaluating hepatic protein metabolism [59]. The increase in ALB is related to a robust immune response in the body [60]. Polysaccharides in *Eucommia ulmoides* Oliver have been shown to have anti-inflammatory effects in mice [34]. In our study, the increased TP and ALB levels in this experiment suggest that ELE can significantly change the body’s protein metabolic efficiency and immunity. Studies have demonstrated that dietary ELE supplementation can regulate serum lipid metabolism and, in detail, inhibit the synthesis of TAG and CHOL in serum and liver, thus reducing lipid accumulation [61,62,63]. In this study, the concentrations of TAG and TC in the serum of the ELE group were significantly reduced, and the concentration of HDL was increased, suggesting that the lipid metabolism of dietary ELE was improved considerably.

As reported, the differential expression of lipid metabolism-related genes in pig muscle tissue leads to differences in meat quality [64]. Our study showed that 0.2% ELE significantly increased MUFA content and decreased PUFA content in muscle. High levels of PUFA in muscle make pork easily oxidized and rancid, resulting in deterioration of processing quality, shelf life, and flavor [65]. Increasing MUFA is beneficial to reduce the hardness of pork, and increasing the dietary MUFA/SFA ratio can also improve human health, for example, by improving β-cell function and reducing insulin resistance [66]. In pigs sensitive to stress responses, the content of C14:0 in the backfat fatty acid composition decreased, and C18:0, C18:2, C18:3, and PUFA were higher, which would deteriorate pork quality [67,68]. In this experiment, the control group exhibited a decreased level of C14:0 and higher levels of C18:0, C18:2n-6, C18:3n-6, and PUFA in muscle compared to the treatment group, indicating a higher sensitivity to stress in the control group. This may be attributed to the reduction in stress levels observed in the treatment group due to ELE supplementation. Further analysis showed that in addition to the decrease in C18:0 content, C24:0 also decreased significantly. Our results indicate that the contribution of C18:0 to SFA is approximately 35%, second only to C16:0. Excessive intake of SFA may be related to the increased risk of chronic diseases such as cardiovascular diseases [69]. The decrease in C18:0 content seems to be beneficial to consumer health. From the perspective of meat appearance and edible quality, C18:1 cis-9 content is positively correlated with meat flavor and overall acceptability, while C18:2n-6 was the opposite [70,71]. In general, an appropriate amount of MUFA intake is beneficial to human health [72]. C18:1cis-9 and C20:1 are considered to help reduce the level of LDL (“bad” cholesterol) [73], and the increase in its content has a positive effect on the cardiovascular health of consumers [74]. Linoleic acid (LA; 18:2n-6) and α-linoleic acid (ALA; 18:3n-3) must be obtained from the diet [75]. They are precursors of n-6 and n-3 PUFA, which can be converted into fatty acids such as C18:2n-6, C18:3n-6, C20:3n-6, and C20:4n-6 in the body. Excessive intake of LA may lead to health problems such as obesity and immunosuppression [76]. According to the World Health Organization/Food and Agriculture Organization of the United Nations recommended standards and human nutritional needs, the ratio of n-6 PUFA/n-3 PUFA in dietary nutrition should be within 5:1 and 10:1. Although n-6 fatty acids are essential for the human body, there is usually an excessive intake of n-6 fatty acids and a relatively low intake of n-3 fatty acids in the daily diet [77]. The decrease in the proportion of n-6:n-3 PUFA is beneficial to human health [78]. Interestingly, the findings of this investigation indicated that ELE inhibited practically all accumulation of n-6 PUFA, including C18:2n-6, C18:3n-6, C20:3n-6, and C20:4n-6, thereby lowering the percentage of n-6 to n-3 PUFA and enhancing the nutritional value of fatty acids in muscle. These results further indicate that dietary supplementation with 0.2% ELE can improve the flavor and quality of muscle fatty acids. We speculate that this is due to the role of ELE in regulating lipid metabolism and anti-inflammation, and the specific mechanism deserves further study.

ELE has been widely reported to have a lipid-lowering function [18,19,79,80]. Our previous work also showed that ELE could reduce fat deposition on the back of finishing pigs [23]. However, it is not clear how ELE affects lipid metabolism in the muscle tissue of finishing pigs. Therefore, in this study, we detected the mRNA levels of lipid metabolism-related genes in both fat and muscle tissues. The results showed that ELE reduced the mRNA level of the ACC gene in dorsal adipose tissue while it upregulated the mRNA expression levels of the ATGL, CPT1B, and FABP4 genes. ACC is the rate-limiting enzyme of fatty acid de novo synthesis [81], ATGL is the main rate-limiting enzyme in TAG hydrolysis [31], and CPT1 is a key rate-limiting enzyme for the entry of long-chain fatty acids (LCFA) into mitochondrial β-oxidation. FABP4 regulates the absorption and intracellular transport of fatty acids [14,82]. These results indicate that ELE can inhibit the excessive accumulation of backfat by regulating some genes in lipid synthesis and degradation pathways [14]. Since the level of lipid oxidation is negatively correlated with protein loss [83], ELE is likely to inhibit backfat thickness and improve the loin-eye area by regulating lipid metabolism in finishing pigs. In muscle tissue, ELE down-regulates the mRNA expression levels of C/EBPα and FAT/CD36 genes. C/EBPα is directly involved in adipocyte differentiation and adipogenesis [84], and its reduced expression may lead to a potential decrease in muscle fat content. In addition, FAT/CD36 can effectively transport LCFA, such as C18:1, C18:2, and C20:4n-6 [85]. Therefore, the changes in the content of PUFA in muscle seem to be related to the decrease in the mRNA expression level of FAT/CD36. Moreover, it has been reported that changes in lipid metabolism greatly affect the composition of the fatty acid profile and amino acid profile in muscle [86]. Interestingly, we did not observe a significant change in IMF content, which may be due to the fact that ELE reduced the β-oxidation of fatty acids in muscle tissue by reducing the mRNA expression levels of CPT1B and ATGL [14,31], thereby maintaining IMF at control levels. In general, we observed the tissue specificity of ELE in regulating lipid metabolism and improved carcass traits and muscle quality by regulating genes related to adipogenesis and lipolysis.

## 5. Conclusions

In conclusion, this study demonstrates that dietary 0.2% ELE supplementation could effectively regulate lipid metabolism and antioxidant capacity of finishing pigs and improve carcass traits, sensory quality, and nutritional value of pork. Although ELE had a powerful lipid-lowering effect, this study provided the first evidence that 0.2% ELE had no negative effect on IMF content, which was achieved by regulating the balance of lipid production and lipolysis in muscle. Therefore, our results provide valuable information for ELE as a natural plant-derived additive for the production of high-quality pork.

## Figures and Tables

**Table 1:** Composition and nutrient levels of basal diets (as-fed basis).

Ingredients (%)	61~90 kg	91~120 kg
Corn	39.70	40.00
Wheat	33.70	39.10
Soybean meal	12.60	6.90
Wheat bran	6.00	6.00
Biological feed	4.00	4.00
Premix ^1^	4.00	4.00
Total	100.00	100.00
Nutrient levels,% ^2^		
Digestible energy, MJ/kg	13.48	13.31
Crude protein	13.94	12.05
Lysine	0.90	0.82
Methionine	0.27	0.26
Threonine	0.61	0.56
Tryptophan	0.15	0.14
Valine	0.54	0.44
Calcium	0.66	0.57
Available phosphorus	0.34	0.26
Fatty acid profile (g/100 g FAME) ^3^		
C14:0	0.05	0.05
C16:0	15.35	15.63
C16:1	0.22	0.23
C18:0	1.65	1.53
C18:1	24.68	24.43
C18:2	54.35	54.49
C18:3	3.70	3.65

^1^ The premix provided the following per kg of diets: 61~120 kg stage, VA 6000 IU, VD_3_ 3000 IU, VE 40 IU, VK_3_ 3 mg, VB_1_ 1.8 mg, VB_2_ 6 mg, VB_6_ 6 mg, VB_12_ 0.024 mg, biotin 4.5 mg, folic acid 0.3 mg, niacin 24 mg, pantothenic acid 20 mg, choline 500 mg, Cu (CuSO_4_·5H_2_O) 15 mg, Fe (FeSO_4_) 100 mg, Mn (MnSO_4_·H_2_O) 100 mg, Zn (ZnSO_4_) 50 mg, I (KI) 0.5 mg, Se (Na_2_SeO_3_) 0.3 mg. ^2^ Digestible energy (DE) was a calculated value, while the others were measured values. ^3^ The concentration of fatty acid classes was expressed as g/100 g, considering 100 g the sum of the areas of all FAME (fatty acid methyl ester) identified.

**Table 2:** Primers used for quantitative real-time PCR.

Genes ^1^	Primer Sequences	Product Length/bp	Acc.Num.
*ACC*	F: 5′-AGCAAGGTCGAGACCGAAAG-3′ R: 5′-TAAGACCACCGGCGGATAGA-3′	208	NM_001114269
*C/EBPα*	F: 5′-GCAGAGATCCCTATAAACCAGC-3′	170	XM_003127015
	R: 5′-TTCAAAGCCCCAAGTCCC-3′		
*HSL*	F: 5′-CACAAGGGCTGCTTCTACGG-3′ R: 5′-AAGCGGCCACTGGTGAAGAG-3′	195	HM591297
*LPL*	F: 5′-CTCGTGCTCAGATGCCCTAC-3′ R: 5′-GGCAGGGTGAAAGGGATGTT-3′	148	NM_214286
*ATGL*	F: 5′-TCACCAACACCAGCATCCA-3′ R: 5′-GCACATCTCTCGAAGCACCA-3′	62	NM_001099930.1
*CPT1B*	F: 5′-GACAAGTCCTTCACCCTCATCGC-3′ R: 5′-GGGTTTGGTTTGCCCAGACAG-3′	176	NM_001007191
*FATP1*	F: 5′-ACCACTCCTACCGCATGCAG-3′ R: 5′-CCACGATGTTCCCTGCCGAGT-3′	208	NM_001083931
*FAT/CD36*	F: 5′-CTGGTGCTGTCATTGGAGCAG-3′ R: 5′-CTGTCTGTAAACTTCCGTGCCTGTT-3′	160	NM_001044622.1
*FABP4*	F: 5′-CAGGAAAGTCAAGAGCACCA-3′ R: 5′-TCGGGACAATACATCCAACA-3′	147	NM_001002817
*PPARγ*	F: 5′-TCGGGACAATACATCCAACA-3′ R: 5′-GACACAGGCTCCACTTTGATG-3′	381	NM_214379
*GAPDH*	F: 5′-CAAAGTGGACATTGTCGCCATCA-3′ R: 5′-AGCTTCCCATTCTCAGCCTTGACT-3′	140	NM_001206359

^1^ *ACC*, acetyl CoA carboxylase; *C/EBPα*, CCAA T/enhancer-binding protein α; *HSL*, hormone-sensitive lipase; *LPL*, lipoprotein lipase; *ATGL*, adipose triacyl glyceride lipase; *CPT1B*, carnitine palmitoyl transferase 1B; *FATP1*, fatty acid transport protein 1; *FAT/CD36*, fatty acid translocase; *FABP4*, fatty acid-binding protein 4; *PPAR γ*, translocase peroxisome proliferator-activated receptor γ; *GAPDH*, reduced glyceraldehyde-phosphate dehydrogenase.

**Table 3:** Effect of dietary ELE supplementation on the growth performance and carcass traits of finishing pigs.

Items ^1^	CON	ELE	SEM	*p*-Value
Initial body weight, kg	75.19	74.38	0.77	0.61
Final body weight, kg	124.66	124.33	1.13	0.89
ADFI, g	2466.3	2480.1	20.4	0.75
ADG, g	824.4	832.5	10.1	0.69
F/G	3.00	2.98	0.03	0.84
Carcass weight, kg	95.50	94.88	0.61	0.63
Carcass yield, %	75.35	76.33	0.45	0.30
Carcass straight length, cm	101.83	99.06	0.89	0.12
Carcass oblique length, cm	85.28	84.24	0.67	0.45
Loin-eye area, cm^2^	28.48	30.02	0.67	0.26
Backfat thickness, cm	2.38	2.16	0.52	0.07
Liver, kg	1.68	1.74	0.48	0.48
Perirenal fat weight, kg	1.58	1.79	1.27	0.42

^1^ CON = control group; ELE = *Eucommia ulmoides* leaf extract supplementation group; ADFI, average daily feed intake; ADG, average daily weight gain; F/G, ratio of feed intake to gain; *n* = 10. Differences were considered statistically significant at *p* ≤ 0.05 and a trend at 0.05 < *p* < 0.10.

**Table 4:** Effect of dietary ELE supplementation on meat quality of *Longissimus dorsi* muscle in finishing pigs.

Items ^1^	CON	ELE	SEM	*p*-Value
After slaughter 45 min				
Lightness (L*)	52.08	48.02	0.90	0.02
Redness (a*)	12.66	12.78	0.17	0.73
Yellowness (b*)	5.40	4.70	0.19	0.07
pH_45min_	5.71	5.98	0.06	0.02
After slaughter 24 h				
Lightness (L*)	57.72	54.78	0.62	0.01
Redness (a*)	13.20	13.57	0.19	0.33
Yellowness (b*)	8.18	8.02	0.23	0.57
pH_24h_	5.30	5.43	0.03	0.03
Pressurization loss, %	29.14	24.19	1.06	0.01
Cooking loss, %	41.49	41.86	0.42	0.66
Drip loss, %	2.74	2.23	0.12	0.03
Shear force, N	69.80	68.33	1.38	0.61
Marbling score	0.90	1.00	0.06	0.58
Meat color score	2.20	3.40	0.24	0.01

^1^ CON = control group; ELE = *Eucommia ulmoides* leaf extract supplementation group; *n* = 10. Differences were considered statistically significant at *p* ≤ 0.05 and a trend at 0.05 < *p* < 0.10.

**Table 5:** Effect of dietary ELE supplementation on *longissimus dorsi* muscle chemical composition of finishing pigs (%).

Items ^1^	CON	ELE	SEM	*p*-Value
Dry matter, %	25.59	25.86	0.17	0.44
Intramuscular fat, %	1.32	1.86	0.18	0.14
Crude protein, %	21.61	22.19	0.19	0.13
Inosine monophosphate, mg/g	2.79	3.30	0.10	0.01

^1^ CON = control group; ELE = *Eucommia ulmoides* leaf extract supplementation group; *n* = 10. Differences were considered statistically significant at *p* ≤ 0.05 and a trend at 0.05 < *p* < 0.10.

**Table 6:** Effect of dietary ELE supplementation on serum biochemical indexes of finishing pigs.

Items ^1^	CON	ELE	SEM	*p*-Value
ALB, g/L	44.31	49.65	1.22	0.02
TP, g/L	73.22	79.23	1.20	<0.01
BUN, mmol/L	3.97	4.40	0.22	0.35
GLU, mmol/L	6.54	6.67	0.31	0.84
TAG, mmol/L	0.70	0.55	0.04	0.06
TC, mmol/L	3.02	2.40	0.11	<0.01
LDL-C, mmol/L	1.60	1.39	0.08	0.10
HDL-C, mmol/L	0.94	1.22	0.06	0.02

^1^ CON = control group; ELE = *Eucommia ulmoides* leaf extract supplementation group; ALB, albumin; TP, total protein; BUN, urea nitrogen; GLU, blood glucose; TAG, triacylglycerols; TC, total cholesterol; LDL-C, low-density lipoprotein cholesterol; HDL-C, high-density lipoprotein cholesterol. *n* = 10. Differences were considered statistically significant at *p* ≤ 0.05 and a trend at 0.05 < *p* < 0.10.

**Table 7:** Effect of dietary ELE supplementation on antioxidant enzyme activities and MDA content of finishing pigs.

Items ^1^	CON	ELE	SEM	*p*-Value
Serum				
GSH-PX, U/mL	122.03	154.56	5.50	<0.01
SOD, U/mL	103.41	149.23	7.76	<0.01
CAT, U/mL	11.00	12.83	0.52	0.08
MDA, nmol/mL	8.96	7.41	0.44	0.08
*Longissimus dorsi* muscle				
GSH-PX, U/mL	144.38	165.87	5.09	0.03
SOD, U/mL	130.99	180.67	7.25	<0.01
CAT, U/mL	14.84	14.88	0.37	0.96
MDA, nmol/mL	11.44	8.00	0.52	<0.01

^1^ CON = control group; ELE = *Eucommia ulmoides* leaf extract supplementation group; GSH-Px, glutathione peroxidase; SOD, superoxide dismutase; CAT, catalase; MDA, malondialdehyde. *n* = 10. Differences were considered statistically significant at *p* ≤ 0.05 and a trend at 0.05 < *p* < 0.10.

**Table 8:** Effect of dietary ELE supplementation on fatty acid composition of *longissimus dorsi* muscle of finishing pigs (%).

Items ^1^	CON	ELE	SEM	*p*-Value
C10:0	0.13	0.14	0.01	0.44
C12:0	0.08	0.09	0.00	0.14
C14:0	1.11	1.35	0.06	0.04
C16:0	24.92	25.47	0.28	0.34
C16:1	2.92	3.27	0.23	0.47
C17:0	0.24	0.18	0.02	0.11
C18:0	15.07	13.97	0.29	0.05
C18:1 trans-9	0.13	0.14	0.00	0.25
C18:1 cis-9	33.43	38.76	1.20	0.02
C18:2n-6	14.46	11.30	0.94	0.09
C20:0	0.16	0.17	0.01	0.54
C18:3n-6	0.15	0.09	0.01	0.04
C20:1	0.54	0.66	0.03	0.05
C18:3n-3	0.46	0.46	0.02	1.00
C20:2	0.35	0.35	0.02	0.93
C20:3n-6	0.62	0.36	0.06	0.02
C20:3n-3	0.08	0.09	0.00	0.83
C20:4n-6	4.66	2.84	0.48	0.05
C24:0	0.35	0.21	0.03	0.04
C22:6n-3	0.12	0.09	0.01	0.11
SFA ^2^	42.07	41.59	0.34	0.50
UFA ^3^	57.93	58.41	0.34	0.50
MUFA ^4^	37.02	42.82	1.38	0.03
PUFA ^5^	20.91	15.56	1.46	0.06
n-3 PUFA ^6^	0.66	0.64	0.03	0.45
n-6 PUFA ^7^	19.89	14.60	1.43	0.06
SFA/PUFA	2.13	2.85	0.24	0.14
n-6/n-3 PUFA	29.62	23.39	1.60	0.05
AI ^8^	0.51	0.53	0.01	0.30
TI ^9^	1.35	1.33	0.02	0.75
h/H ^10^	2.07	2.01	0.04	0.44

^1^ CON = control group; ELE = *Eucommia ulmoides* leaf extract supplementation group; *n* = 10. ^2^ SFA include C10:0, C12:0, C14:0, C16:0, C17:0, C18:0, C20:0, C24:0. ^3^ UFA include C16:1, C18:1 trans-9, C18:1 cis-9, C18:2n-6, C18:3n-6, C20:1, C18:3n-3, C20:2, C20:3n-6, C20:3n-3, C20:4n-6, C22:6n-3. ^4^ MUFA include C16:1, C18:1 trans-9, C18:1 cis-9, C20:1. ^5^ PUFA include C18:2n-6, C18:3n-6, C18:3n-3, C20:2, C20:3n-3, C20:3n-6, C20:4n-6, C22:6n-3. ^6^ n3 PUFA include C18:3n-3, C20:3n-3, C22:6n-3. ^7^ n6 PUFA include C18:2n-6c, C18:3n-6, C20:4n-6. ^8^ AI, atherogenicity index; ^9^ TI, thrombogenicity index; ^10^ h/H, ratio of hypocholesterolemic and hypercholesterolemic fatty acids. Differences were considered statistically significant at *p* ≤ 0.05 and a trend at 0.05 < *p* < 0.10.

**Table 9:** Effect of dietary ELE supplementation on amino acid composition and TAVs of *longissimus dorsi* muscle of finishing pigs.

Items ^1^	Threshold Value (mg/100 g)	Content (mg/100 g)			TAV ^2^			*p*-Value*_(content)_*	*p*-Value*_(TAV)_*
		CON	ELE	SEM	CON	ELE	SEM		
Umami AAs ^3^	/	7.62	8.89	0.42	/	/	/	0.13	/
Glutamic, Glu	30	6.75	7.60	0.35	0.23	0.25	0.01	0.24	0.24
Aspartic, Asp	100	0.87	1.30	0.10	0.01	0.01	0.00	0.02	0.02
Sweet AAs ^4^	/	103.49	119.39	4.02	/	/	/	0.04	/
Glycine, Gly	130	42.13	45.63	2.35	0.32	0.35	0.02	0.48	0.48
Alanine, Ala	60	36.75	46.42	2.49	0.61	0.77	0.04	0.05	0.05
Serine, Ser	150	10.08	11.56	0.38	0.07	0.08	0.00	0.05	0.05
Threonine, Thr	260	14.53	15.78	0.45	0.06	0.06	0.00	0.18	0.18
Proline, Pro	300	6.86	7.31	0.17	0.02	0.02	0.00	0.19	0.19
Lysine, Lys	/	5.25	5.82	0.27	0.10	0.12	0.01	0.31	/
Bitter AAs ^5^	/	56.10	57.08	1.30	/	/	/	0.70	/
Tryptophan, Trp	90	2.67	2.95	0.11	/	/	/	0.23	/
Arginine, Arg	50	11.54	7.19	0.91	0.23	0.14	0.02	0.01	0.01
Histidine, His	20	3.32	4.98	0.34	0.17	0.25	0.02	0.01	0.01
Valine, Val	30	9.73	11.33	0.52	0.24	0.28	0.01	0.13	0.13
Methionine, Met	/	7.76	6.61	0.33	0.26	0.22	0.01	0.09	0.09
Isoleucine, Ile	40	5.74	6.85	0.19	0.06	0.08	0.00	<0.01	<0.01
Leucine, Leu	90	10.9	12.20	0.44	0.06	0.06	0.00	0.13	0.14
Tyrosine, Tyr	/	6.41	7.51	0.21	/	/	/	0.01	/
Phenylalanine, Phe	190	7.13	7.93	0.25	0.08	0.09	0.00	0.11	0.11
Cysteine, Cys	/	2.57	1.66	0.28	/	/	/	0.08	/
Total,	/	190.98	210.6	5.14	/	/	/	0.05	/
EAAs ^6^	/	67.03	74.45	1.56	/	/	/	0.07	/
NEAAs	/	123.95	135.55	3.87	/	/	/	0.07	/

^1^ CON = control group; ELE = *Eucommia ulmoides* leaf extract supplementation group; *n* = 10. ^2^ Taste Activity Value (TAV), TAV = concentration of the component in the sample (mg/100 g)/threshold value (mg/100 g). ^3^ Umami AAs include Glu, Asp. ^4^ Sweet AAs include Gly, Ala, Ser, Thr, Pro, Lys. ^5^ Bitter AAs include Tyr, Arg, His, Val, Met, Ile, Leu, Trp, Phe. ^6^ Essential amino acids include Lys, Ile, Leu, Val, Thr, Phe, Met, His, Trp. Differences were considered statistically significant at *p* ≤ 0.05 and a trend at 0.05 < *p* < 0.10.

## Data Availability

The data supporting the reported results and conclusions can be found in the submitted figure and tables.
